# An Impending Paradigm Shift in Motor Imagery Based Brain-Computer Interfaces

**DOI:** 10.3389/fnins.2021.824759

**Published:** 2022-01-12

**Authors:** Sotirios Papadopoulos, James Bonaiuto, Jérémie Mattout

**Affiliations:** ^1^University Lyon 1, Lyon, France; ^2^Lyon Neuroscience Research Center, CRNL, INSERM, U1028, CNRS, UMR 5292, Lyon, France; ^3^Institut des Sciences Cognitives Marc Jeannerod, CNRS, UMR 5229, Bron, France

**Keywords:** beta bursts, Brain-Computer Interface (BCI), EEG, magnetoencephalography (MEG), motor imagery (MI), neurological rehabilitation, upper limb

## Abstract

The development of reliable assistive devices for patients that suffer from motor impairments following central nervous system lesions remains a major challenge in the field of non-invasive Brain-Computer Interfaces (BCIs). These approaches are predominated by electroencephalography and rely on advanced signal processing and machine learning methods to extract neural correlates of motor activity. However, despite tremendous and still ongoing efforts, their value as effective clinical tools remains limited. We advocate that a rather overlooked research avenue lies in efforts to question neurophysiological markers traditionally targeted in non-invasive motor BCIs. We propose an alternative approach grounded by recent fundamental advances in non-invasive neurophysiology, specifically subject-specific feature extraction of sensorimotor bursts of activity recorded *via* (possibly magnetoencephalography-optimized) electroencephalography. This path holds promise in overcoming a significant proportion of existing limitations, and could foster the wider adoption of online BCIs in rehabilitation protocols.

## Introduction

Central nervous system (CNS) lesions have a major socioeconomic impact on modern societies (World Health Organization, 2011). A grave manifestation involves upper limb motor deficits that affect the quality of life of patients to various degrees depending on both the extent and the exact nature of the lesions. Emerging medical technologies may alleviate these deficits and therefore improve the lives of millions of patients in several ways, diminishing the psychological burden and societal discrimination against people with disabilities.

A prominent direction lies in the development of Brain-Computer Interfaces (BCIs) whose output can be translated into motor commands for rehabilitation protocols (Raffin and Hummel, 2017; Coscia et al., 2019), or devices such as wheelchairs, spellers, exoskeletons, and prostheses (Rosenfeld and Wong, 2017), in order to assist patients in overcoming motor disabilities following stroke, spinal cord injuries, and other CNS pathologies or peripheral deficits, respectively. However, despite the attention that such devices have attracted over the last few years and the fact that numerous research teams are actively working on improving them, to date BCIs have not delivered on their promises. The reasons for this span multiple axes (Chavarriaga et al., 2016) including issues specific to the brain-signal acquisition techniques used, the underlying assumptions about the extracted signals and the targeted mental states, as well as the specific signal processing and feature extraction methods employed.

Broadly, BCIs can be categorized as invasive or non-invasive based on the techniques used to extract relevant brain signals (Daly and Wolpaw, 2008; Hatsopoulos and Donoghue, 2009). Typically, the former require surgery and enable the recording of single- or multi-unit activity, or local field potentials, with depth electrodes or electrocorticography (Hatsopoulos and Donoghue, 2009). The advantages of these techniques are that (a) they allow for close and precise intra- or supracortical placement of the electrodes and thus high spatial resolution and signal-to-noise ratio (SNR), and (b) they can avoid certain sources of noise while still recording high-frequency signals such as neural spiking or high-frequency gamma activity.

Nonetheless, these advantages are blunted by potential risks. As is the case for surgical implantation of electrodes in patients suffering from pharmacoresistant epilepsy or other diseases treated *via* deep-brain stimulation protocols, invasive techniques are inherently accompanied by risk of complications such as infections, subdural and intracerebral bleeding, and brain edema (Hariz, 2002; Wellmer et al., 2012). Moreover, invasive methods based on electrode implantation are characterized by instability over large periods of time due to extensive tissue scaring around the implantation site and electrode drift, which often reduce the numbers of recorded units (Hatsopoulos and Donoghue, 2009). In practice, these drawbacks often require the implanted electrodes to be removed after a fairly short amount of time [although see (Hochberg et al., 2012)].

Considering the risks inherent to surgically implanted electrodes, it is evident that at least for certain applications, non-invasive BCIs seem to be a more viable option for widespread adoption in the foreseeable future, a fact reflected in the predominance of EEG in most BCI applications. Despite the lower SNR and the lower frequency and spatial resolution of extracranially recorded brain signals, advanced signal processing and feature extraction methods (Blankertz et al., 2008; Iturrate et al., 2020), often paired with sophisticated machine and deep learning algorithms (Lotte et al., 2018; Mladenović et al., 2019; Roy et al., 2019), or hybrid BCI designs (Müller-Putz et al., 2015; Hong and Khan, 2017) have steadily improved the decoding capabilities of non-invasive BCIs.

However, most efforts have focused on the development of agent-agnostic algorithms, while only a few studies have focused on refining the neural correlates that are thought to be relevant for a given motor task. We propose that future efforts for online extraction of pertinent brain signals and features should instead concentrate on (a) redefining the neurophysiological markers and features to be extracted and, (b) adopting an individualized and neurophysiologically informed paradigm (Chavarriaga et al., 2016; Mladenović et al., 2019) specifically targeting these markers and features.

Taking advantage of recent work that emphasizes the pitfalls of indiscriminately performing time-frequency analyzes of neural signals (Jones, 2016; Cole et al., 2017; Cole and Voytek, 2017; Donoghue et al., 2021), the importance of transient bursts of frequency-specific activity for behavior (Jones, 2016; Lundqvist et al., 2016, 2018; Sherman et al., 2016; Shin et al., 2017; Lofredi et al., 2018; Torrecillos et al., 2018; Little et al., 2019; Anderson et al., 2020; Khawaldeh et al., 2020; Seedat et al., 2020; Yeh et al., 2020), and the ability to precisely localize these bursts (Bonaiuto et al., 2021), this line of research could vastly improve the decoding capabilities of BCIs and serve as a basis for a wide range of applications that leverage motor-related signals. As a final note, we discuss why we believe non-invasive BCIs should primarily target rehabilitation rather than restoration of control and list a few outstanding questions that remain to be answered.

## Current Approaches to Non-Invasive, Motor Imagery Based Brain-Computer Interfaces

State-of-the-art non-invasive BCI paradigms vary greatly according to the techniques used to extract signals that are potentially informative for a task. Depending on the goal they aim to attain, several setups can exploit different brain signals sometimes even mixed with other, non-brain signals like eye movements and muscle activity (Choi et al., 2017). The recorded brain signals can be electrical, or hemodynamic, measured using electroencephalography (EEG) possibly magnetoencephalography (MEG)^1^, or functional near-infrared spectroscopy (fNIRS), respectively.

Contrary to hemodynamic signals (Nicolas-Alonso and Gomez-Gil, 2012), EEG and MEG signals provide direct measures of neuronal activity and are characterized by high temporal resolution. They are therefore privileged candidates for real-time applications. In this section we will briefly discuss these two techniques as well as the common physiological markers of motor-related cortical activity they record, highlighting the steps usually taken to analyze these signals and their respective shortcomings.

### Electroencephalography and Magnetoencephalography Overview

Electroencephalography measures the electrical activity due to variations of post-synaptic potentials synchronized across large populations of cortical neurons (Buzsáki et al., 2012). The signal is measured as the difference in electric potentials recorded from electrodes relative to a reference (ground) electrode (Nicolas-Alonso and Gomez-Gil, 2012). These post-synaptic potentials also generate weak magnetic fields which are measured by MEG sensors (Hämäläinen and Hari, 2002; da Silva, 2010; Lecaignard and Mattout, 2015).

The major goal of any BCI application is to ultimately enhance and improve the everyday lives of its users. Hence, EEG is frequently the recording technique of choice for BCIs because EEG setups have the advantage of being portable and inexpensive compared to other signal acquisition technologies, thus making them suitable for a wide range of environments from the laboratory to the medical office, or even at home. On the contrary, current SQUID-based MEG systems are more expensive and inherently constrained to magnetic-shielded laboratory environments because of technical limitations imposed by the small amplitude of the measured fields in comparison to multiple sources of noise.

Electroencephalography electrodes are placed on the scalp surface and, as a result, must contend with two main challenges. Firstly, the recorded signals are significantly attenuated and contaminated by physiological and non-physiological noise as they have to traverse multiple layers such as the meninges, cerebrospinal fluid, skull, and muscle tissue before reaching the scalp (Nicolas-Alonso and Gomez-Gil, 2012). Secondly, each electrode samples the activity, distorted by the skull (Lopes da Silva, 2013), of a relatively large cortical area (Krusienski et al., 2011), therefore leading to poor spatial resolution (Lopes da Silva, 2013). Conversely, MEG is not affected to the same extent by either challenge. Although MEG sensors sample magnetic fields from an equivalent cortical area, these fields are only attenuated with distance and not distorted by the presence of layers of tissue and skull between the neural sources and the MEG sensors. As a consequence, MEG offers a higher spatial resolution than EEG. However, in practice it is limited by the head movements that subjects make while performing a task (Medvedovsky et al., 2007), as well as by co-registration errors with anatomical images (Hillebrand and Barnes, 2011). Finally, due to the dynamic nature of brain signals, both EEG and MEG recordings are characterized by great variability, both between individuals, and within recordings of the same individual over long periods of time or different recording sessions.

### Motor Imagery in Electroencephalography and Magnetoencephalography

Brain-Computer Interfaces that aim to leverage motor-related brain activity usually depend on two types of signals. For patients, the signal of interest is occasionally recorded during an attempted movement (Muralidharan et al., 2011). The other common strategy for healthy subjects and patients involves asking subjects to imagine producing movements, known as (kinesthetic) motor imagery (MI) (Pfurtscheller and Lopes da Silva, 1999), on which we will focus hereafter. Whereas a signal recorded while a patient is attempting to perform a movement despite their disability is thought to reflect purely motor functions, MI may also implicate other cognitive processes like mental rotation or conscious access to the motor plan (Jeannerod, 1995; Hanakawa et al., 2003, 2008; Raffin et al., 2012; Meng et al., 2018).

Traditionally, both movement execution and MI have been linked to time-locked changes in induced power within various frequency bands recorded from sensorimotor cortex (Pfurtscheller and Lopes da Silva, 1999; Friston et al., 2006; Ramadan and Vasilakos, 2017). In the beta (∼13–30 Hz) and mu (∼8–12 Hz) frequency bands, these signals are marked by a gradual reduction in power prior to a real or imagined movement, named event-related desynchronization (ERD), followed by a post-movement increase called event-related synchronization (ERS) in the beta band (Pfurtscheller et al., 1996, 2006; Pfurtscheller and Neuper, 1997; Neuper et al., 2006; Tam et al., 2019). In the mu and beta bands, pre-movement ERD has long been considered to reflect neural processes related to movement preparation, initiation and realization, while post-movement ERS was related to the re-establishment of inhibition after the execution of a motor plan (Pfurtscheller et al., 1997; Pfurtscheller and Lopes da Silva, 1999). An ERS is also typically observed in the gamma band (30–120 Hz) around the time of movement onset (Ball et al., 2008; Darvas et al., 2010; Miller et al., 2010), and is thought to reflect movement execution or monitoring (Brovelli et al., 2005; Cheyne and Ferrari, 2013; Gaetz et al., 2013).

### Common Techniques in Motor Imagery Based Brain-Computer Interfaces

Most approaches for MI signal analysis utilize a similar pipeline (Wolpaw, 2002) that involves preprocessing and time-frequency decomposition to extract movement-related ERD and ERS signals, and classification of these signals to predict some aspect of an imagined movement. A great repertoire of signal processing and feature extraction methods have been developed or adapted to particularly address the low SNR and source localization problems inherent in EEG and MEG (Lotte et al., 2018; Iturrate et al., 2020). Signal processing methods aim to improve the quality of the recorded signals by means of artifact rejection, frequency filtering, or spatial filtering. Such methods increase SNR by rejecting non-brain signals, filtering out irrelevant aspects of the signals, and reducing the contribution of non-pertinent areas in the feature extraction step. Dimensionality reduction techniques such as principal component analysis (Zarei et al., 2017) or independent component analysis (Kachenoura et al., 2008) are typically used to select artefactual sources to be removed, or relevant components to be further analyzed (Medeiros de Freitas et al., 2020). Feature extraction refers to the process of extracting the metrics of a signal that is most informative of the imagined movement. Standard procedures include channel selection, and time or spectral feature estimation. For analyzes of frequency-specific changes in power, time-frequency decomposition techniques such as the fast Fourier transform (Herman et al., 2008; Brodu et al., 2011), Hilbert–Huang transform (Wang et al., 2008), or Morlet wavelets (Herman et al., 2008; Brodu et al., 2011) are commonly used.

As the goal of MI based BCI applications is usually to decode aspects of the intended movement from recorded neural activity, the extracted features are used as input to some sort of classifier. Classifiers are trained offline on the sampled activity, yielding a model that maps the underlying features to a set of outputs (e.g., left vs. right imagined movement), and are then employed online to map the recorded activity to an output in real time (Iturrate et al., 2020). Common machine learning algorithms used in BCI applications for decoding a subject’s imagined movement include neural networks (Pfurtscheller and Neuper, 2001; Hazrati and Erfanian, 2010), linear discriminant analysis (Pfurtscheller and Neuper, 2001; Vidaurre et al., 2011; Llera et al., 2014), and support vector machines (Song et al., 2013), while during the last few years the field has experienced a steady shift of interest toward more elaborate deep learning architectures (Roy et al., 2019).

This general BCI pipeline has been widely adopted, but important limitations still exist. Most MI based applications are limited to binary classifications (e.g., left vs. right), or classifications among a few classes at best, and achieve modest results even when continuous paradigms are used, as classification accuracies steeply decrease with an increasing number of output classes (Hong and Khan, 2017). Notably, only a few studies have involved patients, and therefore the real value of BCIs for patients is still unproven. Additionally, despite the sophisticated methods employed, still about a third of subjects have great difficulty or are totally unable to control BCIs, a phenomenon known as BCI illiteracy (Vidaurre and Blankertz, 2010). We argue that this focus on advanced signal-processing and feature extraction techniques for MI based BCIs has effectively rendered ERD and ERS the only signals of interest, creating a gap between the BCI community and recent advances in neurophysiology.

## Proposed Alternative

In light of these considerations we believe that significant progress toward the development of high-fidelity non-invasive BCIs, suitable for online motor decoding, will be achieved only if we attempt to bridge the gap between basic neuroscience and applied neuroengineering. To this end we propose a reassessment of the neurophysiological features of interest for the decoding of motor related activity, and a novel methodology that exploits the advantages of both MEG and EEG in order to take advantage of subtle, individualized physiological markers of motor processing.

### The Quest for Refined Neurophysiological Markers

While standard non-invasive BCI features such as changes in frequency band power are somewhat informative of movement, these approaches discard potentially valuable information in the temporal domain. Time-frequency analyzes rarely take into account the temporal variability and waveform shape of the signals of interest (Donoghue et al., 2021), potentially overlooking the fact that neural activity can occur as bursts rather than oscillations (Jones, 2016; Lundqvist et al., 2016, 2018; Sherman et al., 2016; Shin et al., 2017; Lofredi et al., 2018; Torrecillos et al., 2018; Little et al., 2019; Anderson et al., 2020; Khawaldeh et al., 2020; Seedat et al., 2020; Yeh et al., 2020) and that oscillations can have non-sinusoidal waveform shapes (Cole et al., 2017; Cole and Voytek, 2017). Both of these potentially rich sources of information are lost in typical time frequency analyzes.

**FIGURE 1:**
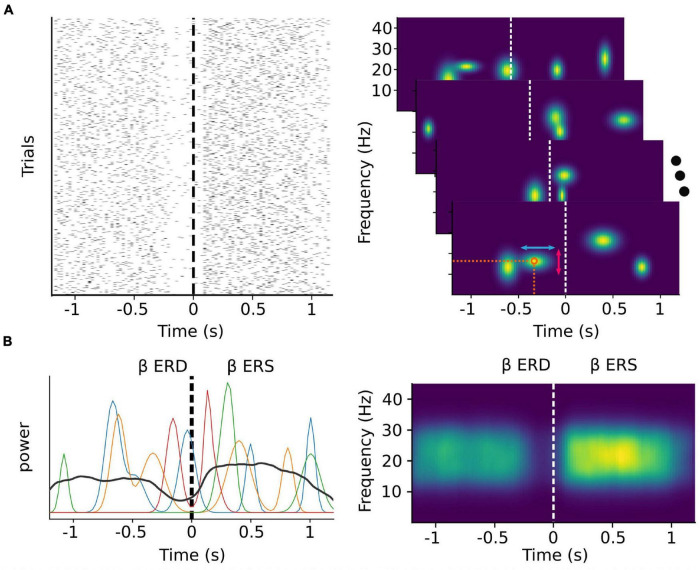
Trial-averaged discrete bursts can appear to be sustained oscillations. Simulated beta burst activity from multivariate Gaussian distributions with a time varying probability and random peak frequency, frequency span, and time duration. **(A)** Left: The timing of simulated bursts in each trial (*N* = 1,000). Right: Time-frequency decomposition analysis of each single trial level (shown for four random ones) allows for the extraction of features such as the exact timing and peak frequency (orange circle), the time duration (red vertical arrows) and the frequency span (light blue horizontal arrows) of each burst. **(B)** Beta band power of the same four random trials from the right panel of **(A)** (colored lines) depicted along with the average beta band power over all trials (black line). During each trial, beta power appears as transient peaks at varying time points. The classically described ERD and ERS phenomena emerge as a consequence of averaging over multiple trials.

In the case of beta activity, it has recently been demonstrated that the majority of sensorimotor activity within this band occurs as discrete transient bursts (Jones, 2016; Lundqvist et al., 2016, 2018; Sherman et al., 2016; Shin et al., 2017; Little et al., 2019; Figure 1A), and that the patterns of pre-movement beta ERD and post-movement beta rebound are a result of averaging these bursts over multiple trials (Little et al., 2019; Wessel, 2020; Figure 1B). Indeed, it has been shown that the timing of beta bursts in motor cortex are, before movement, predictive of response times, and after movement, informative of behavioral errors, more so than changes in beta amplitude (Little et al., 2019). In the right inferior frontal cortex, beta burst timing is related to electromyography correlates of movement cancelation (Hannah et al., 2020), and may coordinate bursts in sensorimotor cortex following successful movement cancelation (Wessel, 2020). Beta burst activity can also be modulated through neurofeedback training (He et al., 2020), making beta burst timing and spatial distribution a potential rich source of information for MI decoding.

**FIGURE 2:**
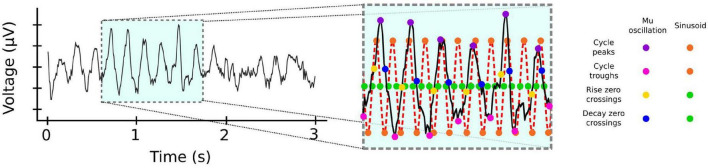
Frequency specific activity can be non-sinusoidal. Mu activity from EEG electrode C4. Inset: Cycle-by-cycle analysis (Cole and Voytek, 2019) of the activity reveals that mu occurs in bursts with significant variability for measures such as peaks (magenta dots) and troughs (pink dots), or rise (yellow dots) and decay (blue dots) duration between cycles, unlike the corresponding measures of a pure 10 Hz sinusoid (orange and green dots). EEG data from BCI Competition IV (Leeb et al., 2007) https://www.bbci.de/competition/iv.

In contrast to beta, activity in the mu frequency band is oscillatory even in single trials (Chen et al., 2021). This activity is typically analyzed using time-frequency decomposition techniques, which assume that the underlying signal is sinusoidal. However, there is now growing consensus that oscillatory neural activity is often non-sinusoidal (Cole and Voytek, 2017, 2019; Donoghue et al., 2021; Fabus et al., 2021), and that the raw waveform shape can be informative of movement (Quinn et al., 2021; Figure 2). Future efforts could take advantage of this possibility by using recently developed non-parametric cycle-by-cycle analyzes (Cole and Voytek, 2019).

### Subject-Specific, Forward Models of Dynamic Activity

The major challenges for non-invasive BCIs are to deal with highly variable and noisy single trial activity, and to overcome the low SNR and limited spatial resolution in order to improve online feature extraction and decoding. We anticipate that these challenges will be even more acute when targeting short-lasting, potentially non-sinusoidal bursts of frequency specific activity. To overcome these challenges, we propose that MEG could be used to complement EEG. Hybrid designs (Allison et al., 2010; Pfurtscheller, 2010) that combine the two techniques yield superior results compared to either technique used alone in certain applications (Corsi et al., 2019; Lecaignard et al., 2021), however such designs are limited to laboratory settings. To deploy a system under more naturalistic settings, one alternative is to use MEG to develop personalized forward models that can subsequently be used to improve the decoding efficacy of online EEG-based BCI applications.

The spatial precision of MEG is traditionally limited by within-session subject movement and between-session co-registration error. However, recently developed high-precision MEG (hpMEG) techniques based on the use of 3D printed individualized head-casts (Meyer et al., 2017), have allowed for fine-grained analyzes of movement-related signals at the level of cortical laminae (Troebinger et al., 2014a,b; Bonaiuto et al., 2018a,b, 2021). Customized forward models based on offline hpMEG analysis could be used to identify the neural features most informative of imaged movements and isolate these features to subsequently optimize EEG-based real-time decoding. In the near future, optically-pumped magnetometers (OPMs) may be able to replace hybrid M/EEG (Boto et al., 2019, 2021; Iivanainen et al., 2019; Roberts et al., 2019; Borna et al., 2020; Hill et al., 2020; Paek et al., 2020; Wittevrongel et al., 2021), as they promise to be more portable and cheaper while also improving SNR.

### A Paradigm Shift Toward Motor Rehabilitation

Lately the BCI community has been drifting away from the restoration of motor control to target motor rehabilitation instead (McFarland et al., 2017; Raffin and Hummel, 2017; Coscia et al., 2019; MacEira-Elvira et al., 2019; Lennon et al., 2020; Micera et al., 2020). Motor rehabilitation is a challenging yet more attainable goal compared to motor restoration, simply because of the amount of complexity that the control of multiple degrees of freedom devices introduces both for the BCI designers and users. Moreover, BCI-assisted rehabilitation protocols are relevant for a wider group of patients beyond those that need to overcome a peripheral handicap; from stroke patients with motor impairments to patients suffering from Parkinson’s disease. The features extracted through bursts analysis could revolutionize these protocols.

As an example, the coupling of this design with mirror therapy based on virtual or augmented reality could be important for a number of reasons. Visual feedback is known to reduce movement errors due to impaired proprioception (Ghez et al., 1995), while it has, also, long been shown in experiments with non-human primates that the role of vision is crucial for the formation of the internal representation of upper limb position, more so than proprioceptive feedback (Graziano, 1999). Additionally, it is established that erroneous visual feedback ultimately results in inaccurate movements being regarded as accurate representations of a subject’s movement intentions (Preston and Newport, 2014). Therefore, hpMEG-optimized BCI training accompanied by digital mirror or camera therapy may soon provide clinicians and, importantly, patients with novel therapeutic solutions.

## Conclusion

Despite the significant effort toward the development of sophisticated algorithms and design paradigms, state-of-the-art EEG-based BCI applications exploiting MI face many limitations. To overcome these limitations, we propose that more subtle neurophysiological markers such as burst timing and waveform shape be explored, and techniques with high temporal and spatial resolution such as hpMEG and OPMs be adopted.

Still, open, outstanding questions related to the proposed paradigm exist. How is burst activity in the motor cortex modulated during imagined rather than performed movements, and how do bursts recorded from different areas of the broader motor network relate to each other? What is the relationship between the continuous nature of movements and burst activity, and how can we exploit it to serve as a reliable marker that will allow us to go beyond discrete classifications? Does the waveform of oscillations change with respect to the recorded area and/or imagined movement, and can it thus give us access to more, complementary features? Importantly, which features of the imagined movements are best explained by this analysis?

We believe that embracing the proposed approach will, in time, provide answers to these questions and significantly improve non-invasive BCIs by extracting richer features of interest from signals recorded with sensitive techniques designed to particularly tackle current limitations.

## Data Availability Statement

The original contributions presented in the study are included in the article/supplementary material, further inquiries can be directed to the corresponding author/s.

## Author Contributions

JB and JM contributed to the conception of the manuscript. SP drafted the manuscript. All authors contributed to manuscript revision, read, and approved the submitted version.

## Conflict of Interest

The authors declare that the research was conducted in the absence of any commercial or financial relationships that could be construed as a potential conflict of interest.

## Publisher’s Note

All claims expressed in this article are solely those of the authors and do not necessarily represent those of their affiliated organizations, or those of the publisher, the editors and the reviewers. Any product that may be evaluated in this article, or claim that may be made by its manufacturer, is not guaranteed or endorsed by the publisher.

## Footnotes

1 The emergence of optically-pumped magnetometers (OPMs) could, unlike currently available systems, render MEG portable and, thus, a very attractive platform for BCI applications in the future.
